# First person – Niranjan Joshi

**DOI:** 10.1242/bio.062531

**Published:** 2026-03-09

## Abstract

First Person is a series of interviews with the first authors of a selection of papers published in Biology Open, helping researchers promote themselves alongside their papers. Niranjan Joshi is first author on ‘ aPKC and F-actin dynamics promote Hippo pathway polarity in asymmetrically dividing neuroblasts’, published in BIO. Niranjan conducted the research described in this article while an undergraduate research assistant in Dr Richard Fehon's lab at The University of Chicago, Chicago, IL, USA. He is now a research technician in the lab of Dr Stefano Di Talia at Duke University, Durham, NC, USA, investigating how development emerges from the dynamic features of molecules, cells and tissues.


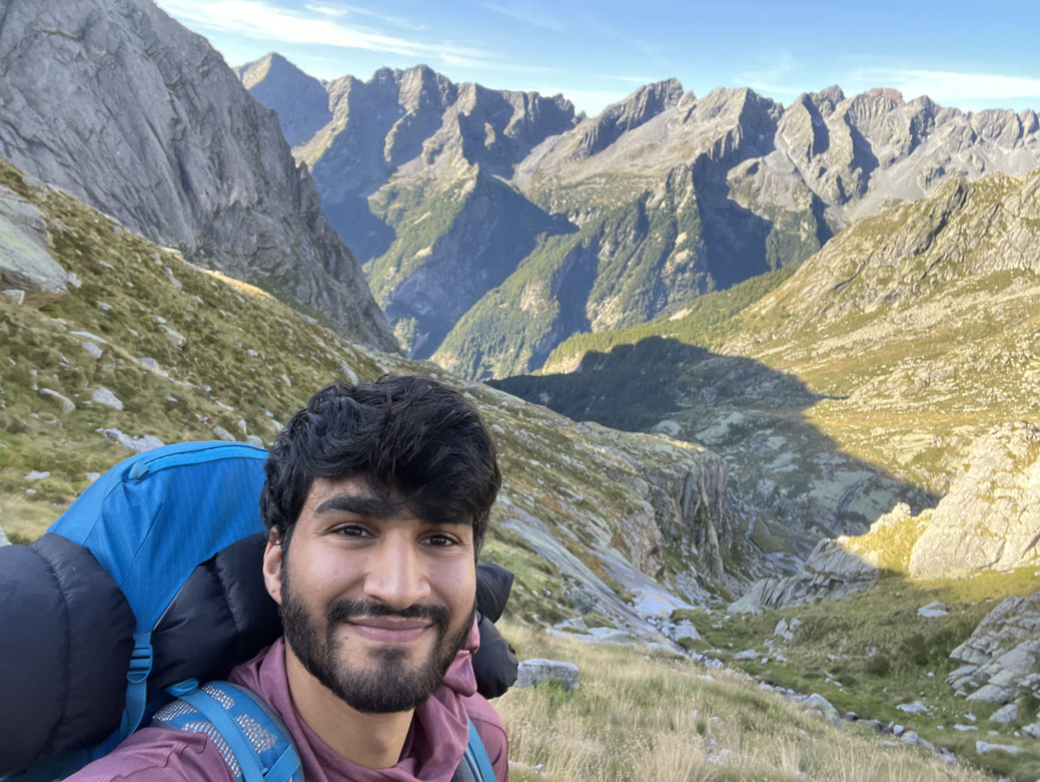


**Niranjan Joshi**

**Describe your scientific journey and your current research focus**

I started college wanting to be a lawyer, but that changed when I took a biology class in my first year. In one of our labs, I imaged the first cell divisions in a nematode zygote. It was so beautiful, and I have been captivated by development ever since. In the subsequent years, I understood development as a dynamic process and one that could be described at many levels of organization. I hope to explore developmental dynamics in graduate school.

**Who or what inspired you to become a scientist?**

Like many biologists, I am endlessly fascinated by the world as it appears through a microscope. I have had wonderful mentors in Rick and Stefano who have nurtured that initial curiosity.

It is such a great time to be a developmental biologist because live imaging is now providing a window into how developmental processes unfold in real time

**How would you explain the main finding of your paper?**

Every organism begins as one cell but ends up being made of many, each of which has a particular identity. How can one cell produce this diversity? One mechanism used is called asymmetric cell division, in which a mother cell divides to produce two unequal daughters. To do so, the mother cell must break symmetry. We study how a molecular signal known to promote asymmetric cell division, the Hippo pathway, breaks symmetry in an asymmetrically dividing stem cell. We found that two Hippo pathway members, Kibra and Salvador, are polarized to one pole of the cell during division. The polarization of Kibra depends on aPKC, an important polarity protein, and the cell's cytoskeleton. We speculate how this polarization could help the cell divide asymmetrically.

**What are the potential implications of this finding for your field of research?**

The Hippo pathway is famous for its role in growth control, but increasing research indicates that it has other important functions. I hope that this study suggests how the pathway could crosstalk with polarity machinery in asymmetrically dividing cells and other polarized contexts.

**Figure:**
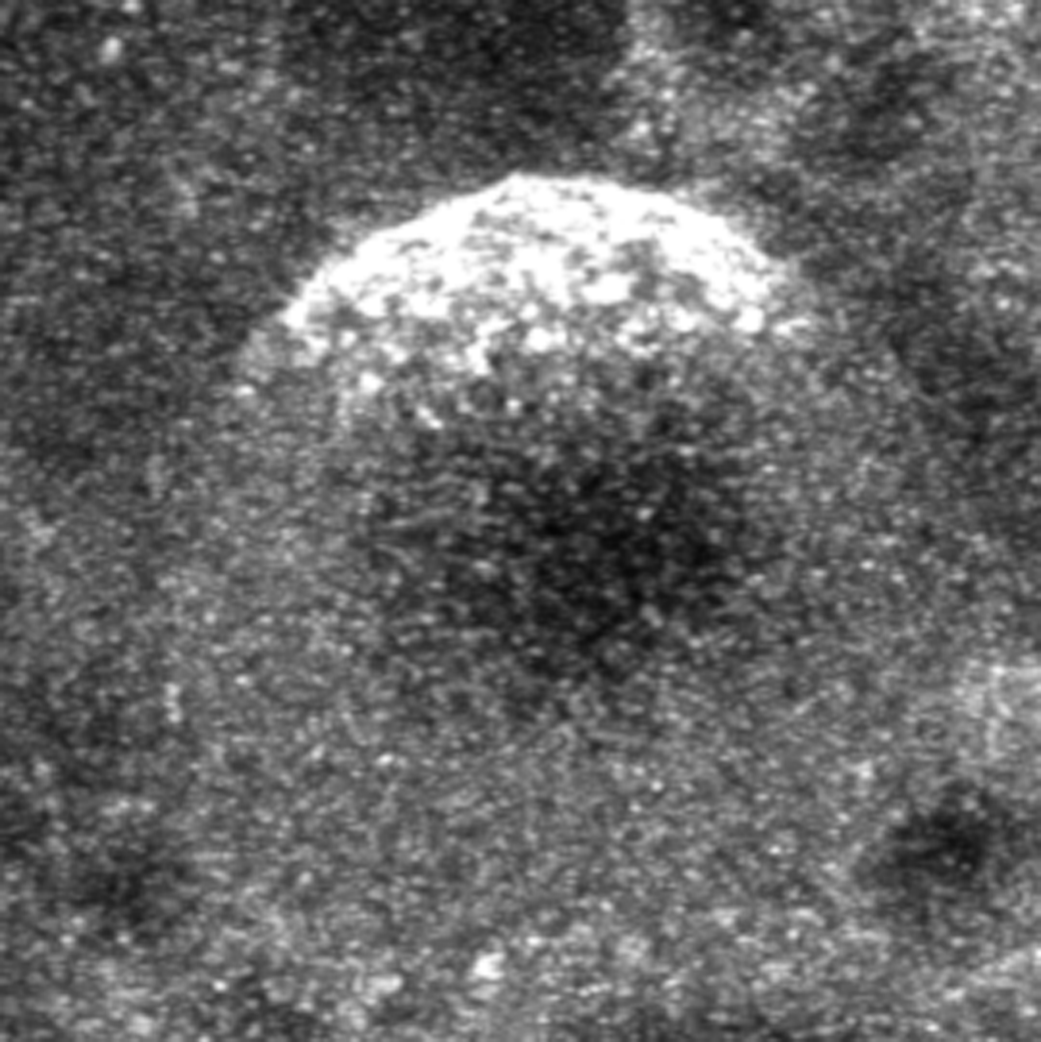
Polarized Kibra in an asymmetrically dividing neuroblast in prophase.

**Which part of this research project was the most rewarding?**

Filming the movies was the best part because asymmetric cell division is spectacular.

**What do you enjoy most about being an early-career researcher?**

It is such a great time to be a developmental biologist because live imaging is now providing a window into how developmental processes unfold in real time. It is exciting to be entering the field right now!

**What piece of advice would you give to the next generation of researchers?**

Let your science be guided by your curiosity and desire to learn. There is always a pressure to win grants and publish, especially as an early-career scientist. These are important but should never be one's sole motivation.

**What's next for you?**

I will begin a doctoral program in quantitative developmental biology this year!
